# Does the SARS-CoV-2 Spike Protein Receptor Binding Domain Interact Effectively with the DPP4 (CD26) Receptor? A Molecular Docking Study

**DOI:** 10.3390/ijms22137001

**Published:** 2021-06-29

**Authors:** Kirsten Cameron, Lina Rozano, Marco Falasca, Ricardo L. Mancera

**Affiliations:** 1 School of Molecular and Life Sciences, Curtin University, GPO Box U1987, Perth, WA 6845, Australia; Kirsten.Cameron@graduate.curtin.edu.au; 2 Curtin Medical School, Curtin Health Innovation Research Institute and Curtin Institute for Computation, Curtin University, GPO Box U1987, Perth, WA 6845, Australia; Lina.Rozano@postgrad.curtin.edu.au (L.R.); Marco.Falasca@curtin.edu.au (M.F.)

**Keywords:** COVID-19, SARS-CoV-2, DPP4, receptor binding domain, molecular interactions

## Abstract

ACE2 has been established as the main receptor for SARS-CoV-2. Since other human coronaviruses are known to use co-receptors for viral cell entry, it has been suggested that DPP4 (CD26) could be a potential additional binding target or co-receptor, supported by early molecular docking simulation studies. However, recent biophysical studies have shown this interaction to be very weak. We have conducted detailed molecular docking simulations to predict the potential binding interactions between the receptor binding domain (RBD) of the spike protein of SARS-CoV-2 and DPP4 and compare them with the interactions observed in the experimentally determined structure of the complex of MERS-CoV with DPP4. Whilst the overall binding mode of the RBD of SARS-CoV-2 to DPP4 is predicted to be similar to that observed in the MERS-CoV-DPP4 complex, including a number of equivalent interactions, important differences in the amino acid sequences of SARS-CoV-2 and MERS-CoV result in substantially weakened interactions with DPP4. This is shown to arise from differences in the predicted proximity, nature and secondary structure at the binding interface on the RBD of SARS-CoV-2. These findings do not support DPP4 being a significant receptor for SARS-CoV-2.

## 1. Introduction

In December 2019, the first cases of a novel coronavirus were reported in Wuhan (China) and since named SARS-CoV-2, which causes the respiratory syndrome COVID-19, characterised by fever, dry cough, breathing difficulties and in severe cases, pneumonia [1]. Phylogenetic analysis of SARS-CoV-2 in early 2020 revealed that SARS-CoV-2 is a *Betacoronavirus*, a type of coronavirus that includes SARS-CoV, MERS-CoV and several bat coronaviruses, including RaTG13 [1]. SARS-CoV-2 shares the same basic structure as the previous seven coronaviruses known to have the ability to infect humans [1]. It is speculated that SARS-CoV-2 is closer to the horseshoe bat coronavirus RaTG13 because it shares over 93% genetic sequence identity, whilst it shares less than 80% sequence identity with SARS-CoV and MERS-CoV [1].

It has been well-established that SARS-CoV binds to the human angiotensin-converting enzyme 2 (hACE2), whilst MERS-CoV binds to human dipeptidyl peptidase 4 (hDPP4 or hCD26) [1,2,3,4,5,6,7]. Experimental studies have established that the spike protein of SARS-CoV-2 can also bind to the ACE2 receptor, and various crystal structures have been reported of the receptor binding domain (RBD) of SARS-CoV-2 in complex with ACE2 [2,3,4,8]. Most studies agree that SARS-CoV-2 binds more strongly to the ACE2 receptor than SARS-CoV does [8].

DPP4 (originally known as lymphocyte cell surface protein, CD26) has been suggested to be a potential cell receptor or co-receptor for SARS-CoV-2 [5,9,10]. The first experimental evidence to support this line of thought is that DPP4 is a known receptor for MERS-CoV [6,7]. Clinical observations have shown that the expression of ACE2 in alveolar type 2 cells (the target of SARS-CoV-2) in the lungs is low, and that DPP4 is one of the top three genes correlated with ACE2 expression, and hence is co-expressed on some of the same cell types in the respiratory tract, which may facilitate viral entry [9,11]. DPP4 is expressed in cells of the lower respiratory tract, fibroblasts of injured skin and lung, muscles, liver, kidneys, prostate, small intestines and activated immune cells [5]. In comparison, the ACE2 receptor is expressed in the lungs, arteries, heart, kidney, intestines and brain [12]. However, cells expressing the ACE2 receptor can be infected by SARS-CoV-2, but not if they express DPP4, suggesting that SARS-CoV-2 does not use DPP4 for viral cell entry [13]. Furthermore, immunofluorescence studies indicated that SARS-CoV-2 did not infect HeLa cells transiently expressing DPP4 [4].

**Figure 1:**
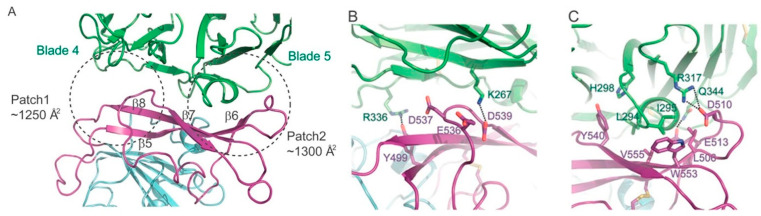
Binding interface of MERS-CoV-DPP4 in the crystal structure of their complex (PDB entry 4L72). (**A**) Overview of the binding regions between DPP4 (green) and MERS-CoV (cyan). The RBM of MERS-CoV (purple) is highlighted. (**B**,**C**) Key DPP4 and MERS-CoV binding residues. Image taken from Wang et al. [6]. Licensed under the Creative Commons Attribution-Non-Commercial-No Derivative Works 3.0 Unported License.

In order to predict the specific potential molecular interactions of SARS-CoV-2 with DPP4, an analysis of the binding interactions between MERS-CoV and DPP4 is pertinent. The binding interactions between MERS-CoV and DPP4 have been characterised through the X-ray diffraction crystal structures of their complex (Figure 1) [6,7]. These structures reveal that residues K267, R336, L294 and I295 in DPP4 are essential for binding [6,7]. In MERS-CoV, the residues Y499 and D539 interact with residues R366 and K267 in DPP4 through hydrogen bonding and salt bridge interactions. Mutagenesis studies that disrupted these interactions by mutating either the MERS-CoV or DPP4 residues resulted in an almost complete loss of binding [6,7]. This effect was also observed when mutagenesis was performed to disrupt the hydrophobic interactions between DPP4 and MERS-CoV, occurring between L294 and I295 in a α-helix of DPP4 and the residues V555, W553 and L506 in MERS-CoV that lie on β-sheets near the loop region in Patch 2 [6,7]. In contrast, the residues R317 and Q344 in DPP4, which form a salt bridge and hydrogen bond with the residues D510 and E513 in SARS-CoV-2, were found to be less significant for binding [6,7].

**Figure 2:**
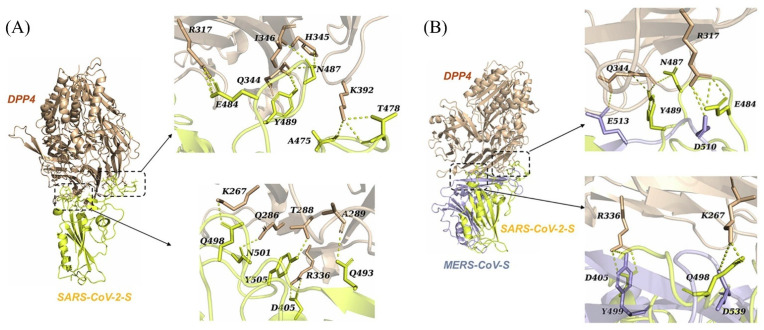
Predicted interactions of the RBD of SARS-CoV-2 with DPP4. (**A**) Key residues predicted in the binding interactions between SARS-CoV-2 (yellow) and DPP4 (wheat). (**B**) Comparison of the residues in (**A**) to the residues in MERS-CoV (purple) known to bind to DPP4. Image taken from Li et al. [5]. Licensed under the Creative Commons Attribution-Non-Commercial-No Derivative Works 4.0 International License.

DPP4 was first modelled by Vankadari and Wilce as a potential receptor for SARS-CoV-2 through the use of molecular docking simulations [14]. No crystal structure of the RBD of SARS-CoV-2 was available at the time and hence the S1/S2 subunits of the viral spike protein were modelled using its genome sequence and SARS-CoV as a template (PDB entry 6ACD) [14]. Subsequent molecular docking studies by Li et al. [5] used the crystal structures of SARS-CoV-2 and DPP4 (PDB entries 6M0J and 4L72, respectively) [3,6]. These simulations used a rigid molecular docking approach with ZDOCK and specific polar residues on DPP4 as restraints, predicting that DPP4 may bind to the RBD of SARS-CoV-2, albeit with a lower binding affinity than for MERS-CoV [5]. SARS-CoV-2 was predicted to bind to DPP4 using a similar binding region to that in MERS-CoV. In general, the same key interactions between DPP4 and MERS-CoV were also predicted between DPP4 and SARS-CoV-2 (Figure 2). Since SARS-CoV-2 and MERS-CoV are related viruses, an important assumption in these studies was that the RBD of SARS-CoV-2 would likely bind to DPP4 in a similar position as MERS-CoV and with a similar conformation [2,3,4,5,6,7]. This assumption was used to identify likely correctly predicted docking poses, in turn guiding the selection of corresponding residues in the RBD of SARS-CoV-2 that were predicted to bind to DPP4 [5].

Recently, it was demonstrated that the spike protein of SARS-CoV-2 does not in fact bind to purified DPP4 (or, more specifically, that it interacts very weakly) [15]. This was shown using surface plasmon resonance (SPR) and ELISA experiments, and was contrasted with the strong interactions of the spike protein of SARS-CoV-2 with the ACE2 receptor [15]. Our molecular docking simulation study seeks to clarify the potential interaction of DPP4 with SARS-CoV-2 and compare it with MERS-CoV.

## 2. Results

### 2.1. Control Molecular Docking Simulations

The binding interactions between the RBD of MERS-CoV and DPP4 predicted by flexible docking using HADDOCK were in agreement with the binding interactions observed in the crystal structure of their complex [6]. A number of new, additional binding interactions were also observed at the binding interface (Supplementary Table S5). This confirmed that HADDOCK was an appropriate molecular docking tool for our purposes. The crystal structures of MERS-CoV and DPP4 were also docked using ZDOCK and their binding interactions were again replicated.

### 2.2. Replication of Previously Reported Molecular Docking Simulations

**Figure 3:**
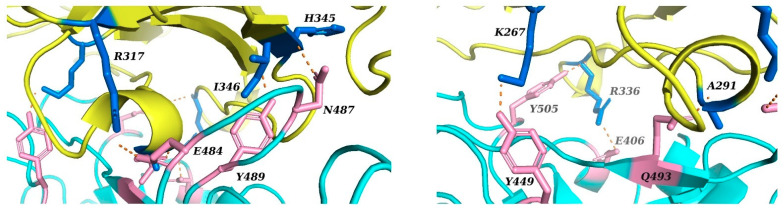
Predicted interactions between the RBD of SARS-CoV-2 and DPP4 obtained using rigid molecular docking with ZDOCK. On the left, the DPP4 amino acid residues R317, I346 and H345 are shown to interact with the SARS-CoV-2 residues E484, Y489 and N487, respectively. On the right, the DPP4 residues K267 and A291 are shown to interact with the SARS-CoV-2 residues Y449 and Q493, respectively. Additionally, R336 of DPP4 interacts with both E406 and Y505 of SARS-CoV-2.

The crystal structures of DPP4 and the RBD of SARS-CoV-2 were docked using a rigid molecular docking approach with ZDOCK. However, it was not possible to replicate the binding interactions between DPP4 and the RBD of SARS-CoV-2 predicted by Li et al. [5]. The binding interactions of the docking pose most similar to the predictions by Li et al. (and indeed having the best interaction energy) are shown in Figure 3. The overall orientation of SARS-CoV-2 appears to have shifted slightly to the right and to the back of the hydrophobic helix of DPP4 compared to the position of MERS-CoV. In comparison to the molecular docking predictions reported by Li et al., this docking pose retains the interactions between the residues R317 and H345 of DPP4 and the residues E484 and N487 of SARS-CoV-2. Interactions that use neighbouring residues to those predicted by Li et al. include the residues K267, R336 and A291 of DPP4, with the residues Y499, E406 and Q493 of SARS-CoV-2. Li et al. predicted that these specific interactions occur between K267 and Q498, R336 and D405, and A289 and Q493 of DPP4 and SARS-CoV-2, respectively. In addition, the following interactions predicted by Li et al. were not reproduced in our simulations: Q344 and Y489, K392 and A475 and T478, Q286 and N501, and T288 and Y505 of DPP4 and SARS-CoV-2, respectively. Overall, the predicted binding interactions bear similarity to the predictions of Li et al. but do not reproduce their findings.

**Figure 4:**
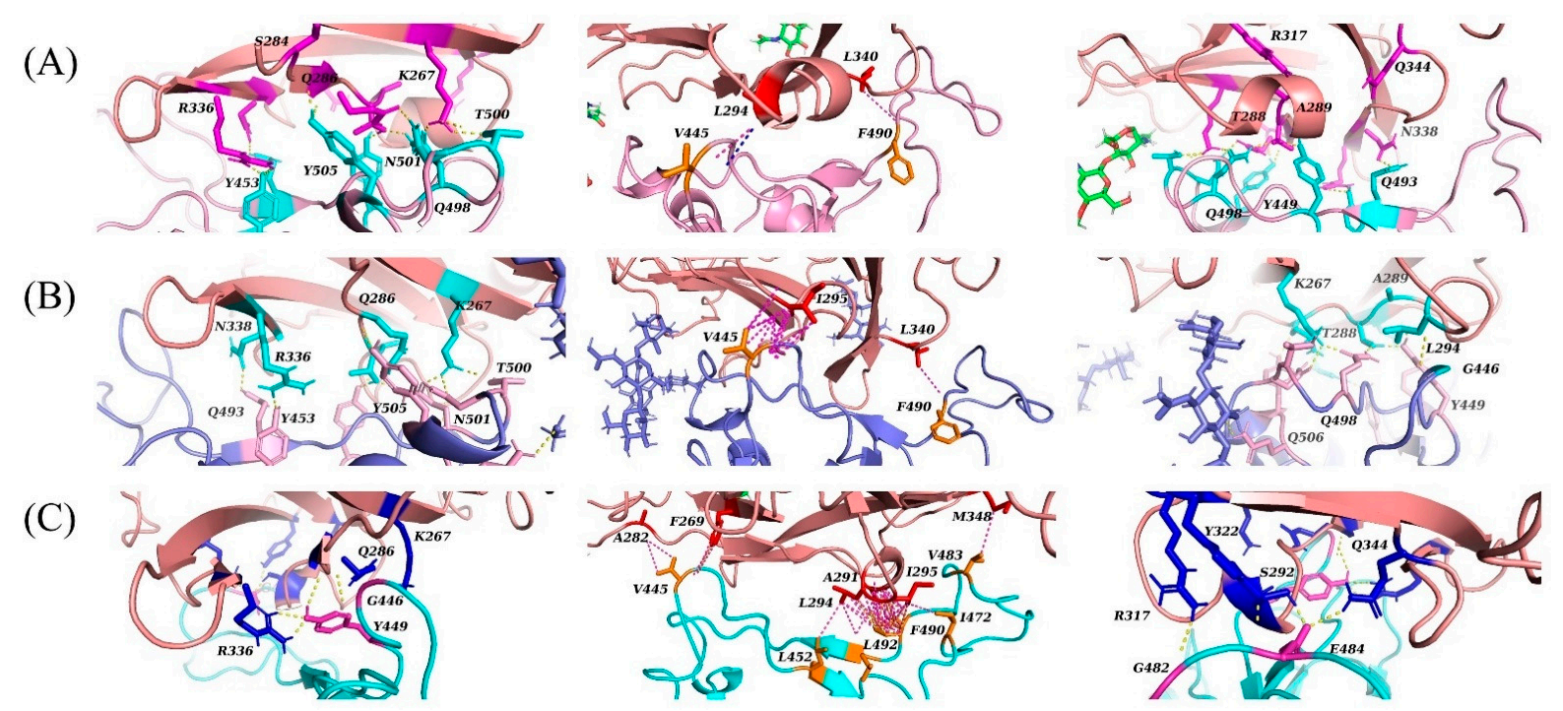
Predicted interactions between the RBD of SARS-CoV-2 and DPP4 obtained using flexible molecular docking with HADDOCK. In all panels, the interacting residues of DPP4 (salmon) and SARS-CoV-2 (pink, purple, cyan) are highlighted. (**A**) Docking variation CS1, (**B**) docking variation CS2 and (**C**) docking variation CS3.

Further attempts were made to replicate these prior predictions using a flexible molecular docking approach with HADDOCK, with three variations in docking restraints (Supplementary Table S4). For each of these docking variations, the best cluster of binding poses was chosen based on the HADDOCK score and considering only docking poses that were visually similar to the binding position of the RBD of MERS-CoV in its crystal structure complex with DPP4 [6]. The binding interactions in the best predictions obtained with each one of these three docking approaches are illustrated in Figure 4.

The first variation in restraints (CS1) aimed to replicate the approach by Li et al. [5] by only specifying the DPP4 residues that these authors defined as key contact residues, to characterise the influence of conformational flexibility. The resulting docking predictions were not consistent with those reported by Li et al. [5] or similar to the interactions that we predicted to occur between DPP4 and SARS-CoV-2 based on the MERS-CoV-DPP4 complex (PDB entry 4L72) [6].

The second variation (CS2) again only specified DPP4 residues but with a different selection (K267, R336, R317, Q344, L294 and I295) [6,7]. As would be expected for similar residue restraints, the resulting docking predictions were similar to those predicted in the above first docking variation (CS1).

The third variation (CS3) specified the DPP4 residues described above and also specified the same SARS-CoV-2 residues as those chosen for the remodelled SARS-CoV-2 structure. This approach aimed to partially remove the bias of the crystal structure of the RBD of SARS-CoV-2 towards its conformation when in complex with the ACE2 receptor [2,3,4]. However, the predictions were poor and inconsistent with all other simulations.

Overall, these molecular docking predictions reveal a tendency for the RBD of SARS-CoV-2 in its crystal structure conformation to avoid the hydrophobic α-helix of DPP4. In comparison to the binding mode of MERS-CoV to DPP4, the binding mode of SARS-CoV-2 in these simulations tended to be skewed such that the RBD would lie behind the hydrophobic α-helix. Compared with MERS-CoV, SARS-CoV-2 lacks available hydrophobic residues that would be able to interact with the α-helix of DPP4 and, instead, has a greater number of polar residues in this region. Hydrophobic interactions tended to form on the opposite side and closer to the centre of the RBD, near the β-sheets of SARS-CoV-2. In the first two docking variations, these hydrophobic interactions tended to form between residue V445 of SARS-CoV-2 and the hydrophobic α-helix of DPP4, and between residues F490 and L340 of SARS-CoV-2 and DPP4, respectively. In the third docking variation, however, residue F490, in addition to I472, L491 and L452 of SARS-CoV-2, was predicted to interact with the hydrophobic α-helix of DPP4. Residue V445 was predicted to interact with the DPP4 residues A282 and F269.

In the MERS-CoV-DPP4 complex, this hydrophobic region of DPP4 is surrounded by the hydrophilic residues R317 and Q344, which interact, respectively, with residues D510 and E513 of MERS-CoV. These residues were not predicted to interact with SARS-CoV-2 in the first two docking variations. In the third variation, however, residue R317 forms a hydrogen bond with G482 (SARS-CoV-2) and Q344 forms a hydrogen bond with E484 (SARS-CoV-2).

In comparison with the binding interactions in the region defined as Patch 1 in the MERS-CoV-DPP4 complex (Figure 1), only the first two docking variations (CS1 and CS2) resulted in a like-for-like substitution of the DPP4 residue R336. In the crystal structure of the MERS-CoV-DPP4 complex, this residue hydrogen bonds to Y499 of MERS-CoV. In our docking predictions, this residue hydrogen bonds to Y453 of SARS-CoV-2, which is the only sequence-aligned residue of SARS-CoV-2 and MERS-CoV to have an interaction to the correct DPP4 residue. This hydrogen bond was not predicted in the third docking variation (CS3). The salt bridge found in the crystal structure of the MERS-CoV-DPP4 complex with residue K267 of DPP4 was instead replaced with hydrogen bonds. In both CS1 and CS2 variations, this residue tended to interact with T500, N501 and Q498 of SARS-CoV-2. In the CS3 variation, K267 of DPP4 is hydrogen-bonded to G446 of SARS-CoV-2. The DPP4 residue Q286 interacted with residues Y505 and Y499 of SARS-CoV-2 in the first and second, and third variation, respectively.

These findings indicate that the molecular simulations were unable to successfully dock the RBD of SARS-CoV-2 with DPP4 in spite of the conformational flexibility that HADDOCK allowed. This suggested that using a different initial conformation of the RBD of SARS-CoV-2 may be necessary, particularly since the conformation used was extracted from its crystal structure in complex with the ACE2 receptor.

### 2.3. Molecular Docking of the Remodelled Structure of the RBD of SARS-CoV-2

**Figure 5:**
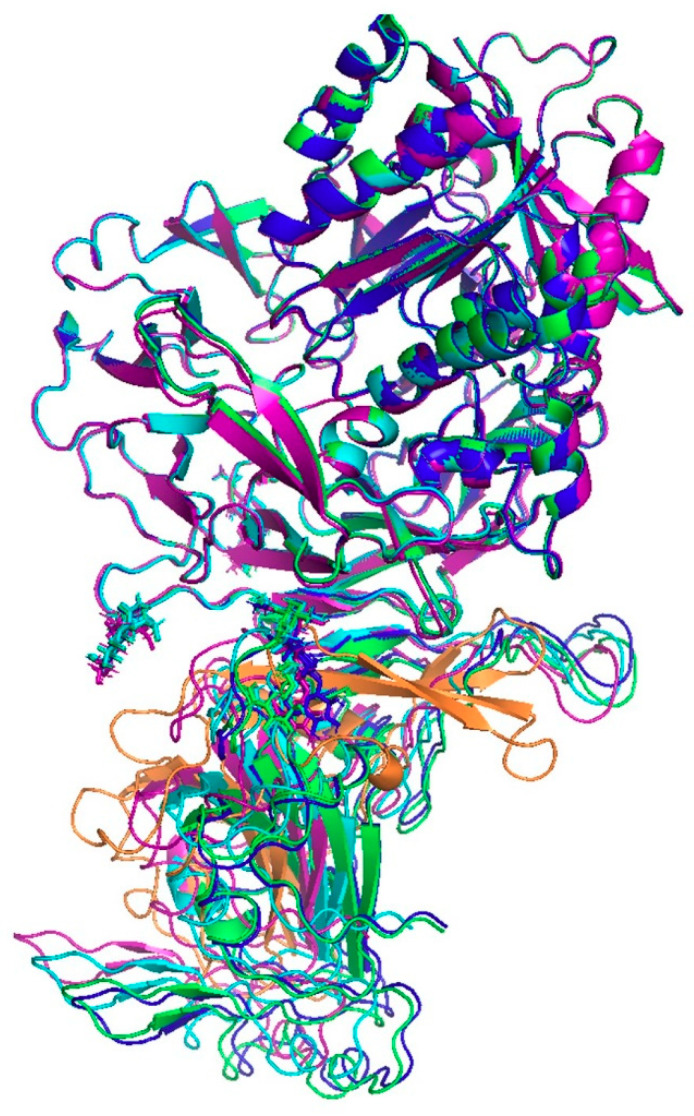
Predicted binding poses between the remodelled structure of the RBD of SARS-CoV-2 and DPP4 obtained using flexible molecular docking with HADDOCK. For comparison, the structure of the RBD of MERS-CoV (orange) is superimposed. Different poses are coloured in cyan, purple, blue and green.

The best clusters of binding poses for the remodelled structure of the RBD of SARS-CoV-2 were selected based on the requirement that docking poses of the clusters should be visually similar to the RBD of MERS-CoV observed in its crystal structure complex with DPP4 [6]. The binding interactions between the RBD of SARS-CoV-2 and DPP4 were analysed to identify the cluster with the binding interactions most similar to those at the binding interface of the MERS-CoV-DPP4 complex and/or to eliminate clusters with unlikely interactions. The best cluster was then selected based upon these interactions and the HADDOCK score (Figure 5). A key difference with respect to the MERS-CoV-DPP4 complex, however, is that the RBD of SARS-CoV-2 was still predicted to be positioned behind the hydrophobic α-helix of DPP4, although to a lesser extent than in the docking predictions using the crystal structure described above.

The best pose selected was ranked favourably by HADDOCK, satisfied interactions with all of the residues in DPP4 specified in the docking restraints and the nature of the interactions with these key residues was reasonable. In comparison to the above-described predicted MERS-CoV-DPP4 complex, the best pose was predicted to have slightly more favourable van der Waals interactions and similar de-solvation energies and buried surface areas (Table 1). However, the electrostatic interaction was substantially less favourable compared to that predicted in the MERS-CoV-DPP4 complex, which is likely due to the substitution of salt bridges with hydrogen bonds for residues R317 and K267 in DPP4. As a consequence, the binding energy of the interaction of the RBD of SARS-CoV-2 with DPP4 is predicted to be substantially less favourable than that of MERS-CoV. The predicted binding energies using the crystal structure of the RBD of SARS-CoV-2 were worse for all three docking variations in restraints (Supplementary Table S6).

Table 1 shows that the binding energy of SARS-CoV-2 to ACE2 is predicted to be somewhat more favourable, reflecting of course not only the different interactions of SARS-CoV-2 with each receptor, but also the differences in receptor structures. The de-solvation energy was lower for the SARS-CoV-2-ACE2 complex; however, the van de Waals and electrostatic energies and buried surface area were predicted to be higher than those predicted for the SARS-CoV-2-DPP4 complex.

**Figure 6:**
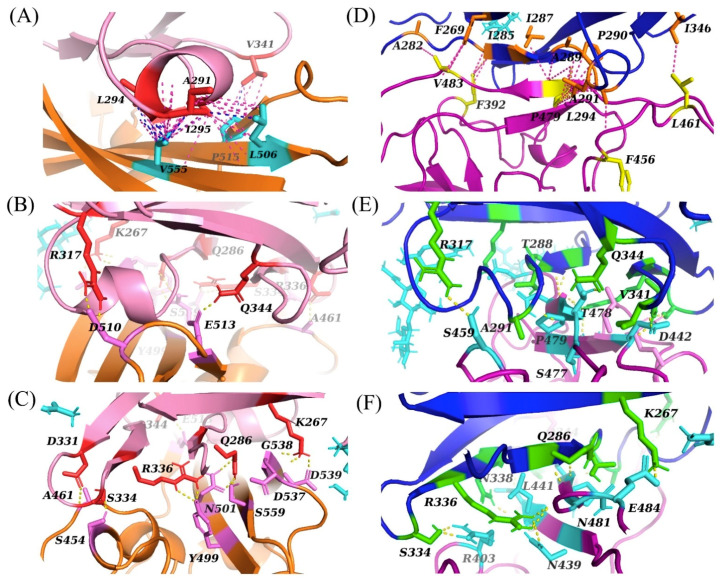
Predicted interactions between the RBD of MERS-CoV and remodelled SARS-CoV-2 with DPP4 obtained using flexible molecular docking with HADDOCK. (**A**–**C**) Binding interactions between MERS-CoV (orange) and DPP4 (pink). (**D**–**F**) Binding interactions between the remodelled structure of SARS-CoV-2 (purple) and DPP4 (blue). Hydrophobic interactions (magenta and blue) in both complexes are shown in panels (**A**,**D**). Polar interactions (yellow) to the right of the hydrophobic α-helix are described in panels (**B**,**E**), and to the left of the hydrophobic α-helix are described in panels (**C**,**F**).

Analysis of this best docking pose revealed that the binding interactions between the remodelled structure of the RDB of SARS-CoV-2 and DPP4 are comparable to the binding interactions in the predicted MERS-CoV-DPP4 complex (Figure 6) and those observed in the MERS-CoV-DPP4 crystal structure (Figure 1). Figure 6 also shows that the docking predictions for the MERS-CoV-DPP4 complex are very similar to the corresponding crystal structure. Consequently, the crystal structure of the MERS-CoV-DPP4 complex was used to compare the docking predictions for the SARS-CoV-DPP4 complex described below.

The SARS-CoV-2-DPP4 complex was predicted to exhibit hydrophobic interactions with a hydrophobic α-helix in DPP4 [6,7]. In contrast to MERS-CoV, however, residues L294 and A291 in DPP4 were predicted to interact with residues P479 and F456 in SARS-CoV-2 [6,7]. Additionally, P479 interacts with residues I287, A289 and P290 in DPP4. Unlike in the MERS-CoV-DPP4 complex, these hydrophobic interactions do not include I295 in DPP4, and the residues in SARS-CoV-2 do not surround the hydrophobic residues in DPP4 as the residues in MERS-CoV do [6,7]. Initially, the residue L461 in SARS-CoV-2 was expected to interact with residues L294 and I195 in DPP4; however, we predicted L461 to instead form a hydrophobic interaction with I346 in DPP4 [6,7]. Furthermore, residues A282, F269 and I285 in DPP4 were predicted to have hydrophobic interactions with residues V483 and F392 in SARS-CoV-2 [6,7]. The latter were not conserved in all of the docking poses of this cluster.

SARS-CoV-2 was also predicted to exhibit polar interactions with positively charged residues R317 and Q344 in DPP4 that surround its hydrophobic α-helix [6,7]. In the MERS-CoV-DPP4 complex, these residues interact with negatively charged acidic amino acid residues; however, in the predicted SARS-CoV-2-DPP4 complex, these residues form hydrogen bonds with S459 and T478 [6,7]. As the salt bridge interactions present in the MERS-CoV-DPP4 complex are replaced with only hydrogen bonding, these interactions are arguably much weaker [6,7]. However, mutagenesis studies found that these interactions were not significant for the binding of MERS-CoV to DPP4 [6,7]. In addition, new hydrogen bonding interactions were predicted between the backbones of residues A291, T288 and V341 in DPP4 and the side chains of residues S477, P479 and D442 in SARS-CoV-2. The interaction between T288 in DPP4 and P479 in SARS-CoV-2 was not conserved in all of the other docking poses of this cluster.

The interactions between SARS-CoV-2 and DPP4 within the second region encompassing polar interactions were found to be reasonable (but weaker) substitutes for the interactions seen in the MERS-CoV-DPP4 complex, in which hydrogen bonding and salt bridges with the amino acid residues R336 and K267 in DPP4 were found to be essential for binding [6,7]. In the predicted SARS-CoV-2-DPP4 complex, R366 in DPP4 forms hydrogen bonds with two asparagine residues, N439 and N481. The latter interaction is conserved across half of the four poses, whereas the former interaction is only observed in the best docking pose. This interaction is, however, often observed in other clusters. It is likely that R336 in DPP4 interacts with N481 on the second β-sheet of SARS-CoV-2 because a neighbouring arginine on SARS-CoV-2, R403, repeals R366 in DPP4 to orient this residue closer to a second β-sheet. Residue K267 in DPP4, on the other hand, interacts with residue E484 in SARS-CoV-2. This interaction is deemed to be reasonable as the interaction with an aspartic acid in the MERS-CoV-DPP4 complex is predicted to be replaced with an interaction with a glutamic acid [6,7]. Further polar interactions were predicted between residues S334, Q286 and N338 in DPP4 and residues R403, N481 and L441 in SARS-CoV-2. The hydrogen bond between the side chain of N388 and the backbone of L441 is unlikely, however, because leucine has a tendency to interact with other non-polar residues. This interaction was only observed in this docking pose.

Furthermore, these interactions can be compared to those observed in the complex DPP4 forms with adenosine deaminase (ADA). Interestingly, there is a significant cross-over between the DPP4 residues that Weihofen et al. [16] discuss in the ADA-DPP4 complex and those observed in the predicted interactions in the MERS-CoV-DPP4 and SARS-CoV-2-DPP4 complexes (the latter using the remodelled structure of SARS-CoV-2).

In terms of polar interactions, Weihofen et al. [16] highlight E139 and D143 in ADA as important residues for binding to DPP4. The residue E139 in ADA hydrogen bonds with S292, A291, P290 and Q344 in DPP4. Hydrogen bonding is also observed between D143 and Q344 of ADA and DPP4, respectively. Interestingly, in the MERS-CoV-DPP4 complex, Q344 in DPP4, one of the polar residues that surrounds the hydrophobic α-helix, hydrogen bonds to E513 in MERS-CoV [6,7]. In our remodelled structure, Q344 hydrogen bonds to T478 in SARS-CoV-2. In the MERS-CoV-DPP4 complex, R317 is highlighted as an important residue that forms a salt bridge with the MERS-CoV residue D510 [6,7]. In our predictions with the remodelled structure, this interaction is replaced with hydrogen bonding to S459; however, it is absent in the ADA-DPP4 complex. This suggests that the DPP4 residue Q344 may be of greater importance when DPP4 binds to other proteins.

Furthermore, the ADA residue D127 forms a salt bridge to R336 in DPP4. In the MERS-CoV-DPP4 complex, R336 was identified as an important polar residue that hydrogen bonds to the MERS-CoV residue Y499 [6,7]. Using the remodelled SARS-CoV-2 structure, R336 was predicted to hydrogen bond with N439 and N481 of SARS-CoV-2. As ADA is able to form a salt bridge with the DPP4 residue R336, this likely improves the binding energy in the ADA-DPP4 complex and again confirms that R336 is an important residue when DPP4 binds to other proteins. It should be noted that a salt bridge was observed between K267 and D539 in DPP4 and MERS-CoV, respectively [6,7]. The same interaction was predicted to occur between K267 and E484 in DPP4 and the remodelled SARS-CoV-2, respectively. However, no interaction with K267 in DPP4 is observed in the ADA-DPP4 complex. This suggests that while an interaction with K267 is not necessary for binding to DPP4, strong polar interactions in this area do increase binding to DPP4.

Additional polar interactions in the ADA-DPP4 complex include those between A289 and K80 of DPP4 and ADA [16]. Hydrogen bonding to this DPP4 residue is not predicted with the remodelled SARS-CoV-2 structure (however, it is in the C1 docking variation); however, the neighbouring DPP4 residue A291 interacts with the remodelled SARS-CoV-2 residue S477. Weihofen et al. [16] also describe the hydrogen bonding between the DPP4 residues T288 and Q286 and the ADA residue D77. Hydrogen bonding with DPP4 residue Q286 was not observed in the crystal structure of the MERS-CoV-DPP4 complex; however, it was predicted to hydrogen bond to N501 and S559 in MERS-CoV as an additional interaction in our control docking predictions. Hydrogen bonding occurs between T288 and P479, and Q286 and N481, of DPP4 and SARS-CoV-2, respectively. The substitution of non-charged polar residues in the place of the charged ADA residues and hydrogen bonding to proline (the SARS-CoV-2 residue P479) is unlikely to impact binding to DPP4.

Weihofen et al. [16] reported a number of hydrophobic interactions across the binding interface in the ADA-DPP4 complex. In the ADA-DPP4 complex, the hydrophobic α-helix (DPP4 residues I295, L294, A291 and S292) interacts with Y84 and R81 of ADA. In contrast to ADA, the DPP4 residues I295 and L294 interact with the MERS-CoV residues V555, W553 and L506. Our remodelled structure, discussed above, predicts that L294 and A291 hydrophobically interact with the SARS-CoV-2 residues P479 and F456. The DPP4 residue V341 was also identified as important for binding in the ADA-DPP4 complex [16,17]. This residue, along with Q344, was reported to hydrophobically interact with the ADA residues E139 and Q138. These interactions are not present in the MERS-CoV-DPP4 crystal structure; however, hydrophobic interactions were predicted in the docking control for V341 and P515 in DPP4 and MERS-CoV, respectively. No hydrophobic interactions were predicted between V341 and Q344 in DPP4 and the remodelled SARS-CoV-2, respectively; however, these DPP4 residues were involved in hydrogen bonding to SARS-CoV-2.

Both Weihofen et al. [16] and Abbott et al. [17] agree that hydrophobic interactions are important for binding through the observation that the ADA-DPP4 complex dissociates at low ionic strength and the point mutations L294R and V341K in DPP4 resulted in a loss of binding. This reflects our conclusions that the inability of SARS-CoV-2 to effectively interact with the hydrophobic α-helix in DPP4 is detrimental to binding.

Additional hydrophobic interactions were reported between the DPP4 residues S292, A291 and P290, and the ADA residue E139 [16]. These interactions are not observed in the MERS-CoV-DPP4 crystal structure [6,7]. However, in the remodelled SARS-CoV-2, the DPP4 residues L294, A291, P290 and A289 hydrophobically interact with P479 in SARS-CoV-2. In the ADA-DPP4 complex, the DPP4 residue I346 was reported to interact with the ADA residues D143 and R142. Interactions with I346 were again not observed in MERS-CoV-DPP4; however, this residue interacts with L461 in SARS-CoV-2. This suggests that, in addition to the residues in the hydrophobic α-helix, the DPP4 residues V341 and I346 should be considered in future predictions of interactions with other proteins.

## 3. Discussion

The molecular docking simulations reported here indicate that predicting the structure and interactions of SARS-CoV-2 with DPP4 requires remodelling of its structure. Using the crystal structure of the RBD of SARS-CoV-2 in complex with the ACE2 receptor imposes a bias towards the conformation that the protein has when it is in complex with the ACE2 receptor [2,3,4,5]. Furthermore, the RBD of SARS-CoV-2 consists predominantly of loop regions that likely exhibit a high degree of conformational flexibility [2,3,4]. Consequently, remodelling of the RBD of SARS-CoV-2 may be necessary to make it adopt a conformation at its interface closer to that observed in the interaction of MERS-CoV with DPP4. It is important to note that the remodelled structure of SARS-CoV-2 was chosen from multiple models on the basis of its similarity to the structure of MERS-CoV at the binding interface [6,7]. Whilst this was an informed decision aimed at reducing steric clashes with DPP4, it remains an arbitrary choice that may have had a substantial impact on the predicted binding poses and binding affinities. The energy barrier that is associated with this conformational change was not characterised here, but it could be large and unfavourable given the increase in β-sheet content in the relevant loop region. Furthermore, this remodelled structure may not necessarily reflect the actual conformation that the RBD of SARS-CoV-2 could adopt during a potential interaction with DPP4.

Both rigid and flexible molecular docking simulations with ZDOCK and HADDOCK, respectively, were unable to reproduce the predictions of Li et al. [5]. In comparison to this and other previous molecular docking studies that attempted to dock the RBD of SARS-CoV-2 with DPP4, the potential binding of SARS-CoV-2 to DPP4 was predicted to potentially involve all or a combination of residues R336, K267, R317, Q344, Q286, A289, A291, I295 and L294 in DPP4 [5,6,7,14]. Our docking simulations predict that hydrogen bonding and salt bridge interactions could occur between R336 and N481, K267 and E484, R344 and T478 and between R317 and S459, in DPP4 and SARS-CoV-2, respectively. In addition to these polar interactions, potential hydrophobic interactions are predicted between residues P479, L461 and V483 in SARS-CoV-2 and residues L294, P290, I287, I346 and F269 in DPP4.

The binding energy between SARS-CoV-2 and DPP4 was predicted to be substantially less favourable than that of the confirmed interaction between MERS-CoV and DPP4. This suggests that whilst it may be possible to make a computational prediction of the interactions between DPP4 and SARS-CoV-2, the interaction is much weaker than with MERS-CoV. This is likely because all of the docking approaches used indicated that the RBD of SARS-CoV-2 is unable to interact effectively with the key hydrophobic α-helix of DPP4, leading to a necessary shift in the location of the RBD compared to the interaction of MERS-CoV with DPP4. Compared with the RBD of MERS-CoV, SARS-CoV-2 does not possess hydrophobic residues that can interact in the same manner with the α-helix of DPP4. It possesses instead a larger number of polar residues that are unfavourable for interaction with this hydrophobic region in DPP4.

A recent biophysical study revealed that the spike protein of SARS-CoV-2 does not bind to DPP4, or at best it binds very weakly, whilst also confirming that MERS-CoV and SARS-CoV can bind strongly to DPP4 and ACE2, respectively [15]. These differences in binding were rationalised in terms of the fact that the RBD of SARS-CoV-2 has a greater hydrophobic surface than the RBD of MERS-CoV, but the loop region at the interface was predicted to prevent the underlying hydrophobic residues from interacting with the hydrophobic regions on DPP4 [15]. In addition, negatively charged residues at this region were predicted to repeal the hydrophobic region of DPP4 [15]. Furthermore, the RBD of SARS-CoV-2 lacks polar or charged residues, whereas there are charged or polar residues in MERS-CoV that can bind to similar residues in DPP4 (see Figure 5 in [15] for a comparison of the protein binding surfaces of DPP4, adenosine deaminase, MERS-CoV RBD and SARS-CoV-2 RBD). This is consistent with our molecular docking predictions.

It is important to note that the interactions predicted in this docking study would likely be impacted in some of the new variants of SARS-CoV-2 that emerged in late 2020, such as those from the UK, South Africa, Brazil and India [18]. The UK variant (B.1.1.7 or Alpha) has the mutation N501Y in the RBD of SARS-CoV-2 [18]. This mutation is also present in the South African (B.1.351 or Beta) and Brazilian (P.1 or Gamma) variants, which also have the mutations K417N/T and E484K. The Indian (B.1.617.2 or Delta) variant has different mutations in the RBD of SARS-CoV-2: L452R and T478K. Whilst the N501Y and K417N/T mutations are known to favour the binding of SARS-CoV-2 to the ACE2 receptor [1,2,3], this docking study suggests that only N501Y, E484K and T478K are relevant to the best-predicted binding modes between SARS-CoV-2 and DPP4. In the first two docking variations using the crystal structure of the RBD of SARS-CoV-2 (C1 and C2), the residue N501 of SARS-CoV-2 was predicted to interact with K267 of DPP4. Whilst a tyrosine residue in the N501Y mutation would still be able to hydrogen bond to K267 of DPP4, the presence of the bulky aromatic ring may cause some deleterious changes to this interaction. In the third docking variation (C3), the residue E484 of SARS-CoV-2 is predicted to interact with residue Q344 of DPP4. As glutamine is both an H-bond donor and acceptor, interaction with this residue could be replaced by lysine in the E484K mutation, although the longer side chain length might also be deleterious. In contrast, the residue E484 is predicted to interact with residue K267 of DPP4 when the remodelled structure of SARS-CoV-2 is used. In this case, the substitution of the negatively charged glutamate with a positively charged lysine in the E484K mutation would lead to a repulsive interaction with K267 of DPP4. This would significantly impact the docking predictions using the remodelled structure of SARS-CoV-2, as residue K267 of DPP4 is assumed to be a key residue for binding. In the Delta variant, the SARS-CoV-2 residue L452 interacts with L294 in DPP4 in the C3 docking variation. A L452R mutation would interfere with this hydrophobic interaction; however, in the remodelled SARS-CoV-2 structure (our best prediction), the L452 residue is far from the binding interface and hence the mutation would have no impact. Finally, the mutation T478K would likely have a negligible impact on binding. The substitution with a positively charged side chain in the place of a polar neutral threonine side chain would still allow hydrogen bonding to the DPP4 residue Q334. In the MERS-CoV-DPP4 crystal structure, a negatively charged glutamic acid residue hydrogen bonds to Q334 [6,7]. Overall, the key mutations observed in the above new variants of SARS-CoV-2 would not make the interaction with DPP4 more likely.

## 4. Materials and Methods

The interactions between the RBD of SARS-CoV-2 and DPP4 were predicted based on the use of different conformations of the protein and different selections of interacting residues to guide a flexible molecular docking simulation approach. The first selection of residues in SARS-CoV-2 was obtained by substituting the interactions made by corresponding residues in MERS-CoV determined upon alignment and spatial positioning of the crystal structure of the RBD of SARS-CoV-2 to that of MERS-CoV (in complex with DPP4). Visualisation of the overlaid crystal structures of SARS-CoV-2 and MERS-CoV revealed, however, the presence of multiple steric clashes between SARS-CoV2 and DPP4. To resolve this, molecular docking simulations with conformational flexibility were performed using both the original crystal structure of the RBD of SARS-CoV-2 (taken from its complex with the ACE2 receptor) and a remodelled structure based on the structure of MERS-CoV bound to the DPP4 receptor.

### 4.1. Selection and Preparation of X-ray Crystal Structures

The RBD of the spike protein of SARS-CoV-2 was used in the docking simulations. This structure has been reported in complex with the ACE2 receptor [2,3,4]. Entry 6M0J (with the highest resolution of 2.45 Å) was chosen from the available crystal structures of this complex in the Protein Data Bank (PDB) [2,3,4]. This structure was docked to that of DPP4 taken from its crystal structure in complex with the RBD of MERS-CoV (entry 4L72) [6]. The glycans in DPP4 at the MERS-CoV-DPP4 binding interface (chains C, D and E) were retained as they could influence the molecular docking predictions. No glycans are present in the RBD of SARS-CoV-2. All crystal structures and the remodelled structure of SARS-CoV-2 (see below) had hydrogen atoms added and were energy-minimised for 200 steps with the generalised Born implicit solvation model in BIOVIA Discovery Studio (Dassault Systèmes, Vélizy-Villacoublay, France) prior to docking.

### 4.2. Selection of Binding Residues in the RBD of SARS-CoV-2

Based upon the X-ray structure of the MERS-CoV complex with DPP4, the amino acid residues K267, R336, R317, Q344, L294 and I295 in DPP4 were selected as likely interacting residues with SARS-CoV-2 [6,7]. The crystal structure and the remodelled structure (see further below) of SARS-CoV-2 were visualised and overlaid onto the structure of the MERS-CoV-DPP4 complex (PDB entry 4L72) [6] in PyMOL 2.0 (Schrodinger, New York, NY, USA) using the *super* and *extra_fit* functions.

The residues of SARS-CoV-2 first considered as the desired interacting residues were selected based upon the sequence and structural alignment of the remodelled SARS-CoV-2 structure to MERS-CoV in complex with DPP4 (PDB entry 4L72) [6] (see Supplementary Figures S1 and S2). To identify SARS-CoV-2 residues that could form potential interactions with the above selected DPP4 residues, the regions around these key DPP4 residues were examined. SARS-CoV-2 residues that were in close proximity to these DPP4 residues and either matched the corresponding MERS-CoV residues or were of a similar type (non-polar, polar or charged) were selected. The same SARS-CoV-2 residues that were chosen for the remodelled structure were also chosen for the unmodified crystal structure.

### 4.3. Remodelling of the Structure of the RBD of SARS-CoV-2

**Figure 7:**
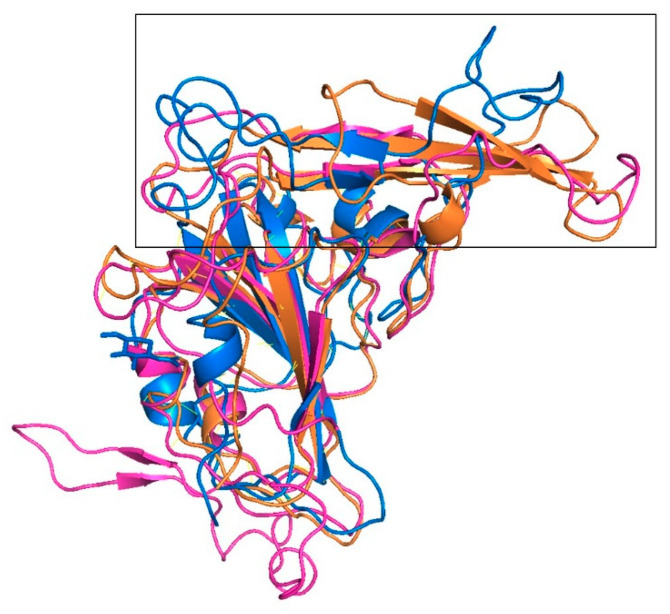
Superimposition of the structures of the RBDs of MERS-CoV and SARS-CoV-2. In comparison to the crystal structure of MERS-CoV (orange), the β-sheets in the crystal structure of SARS-CoV-2 (blue) are significantly shorter with a loop region orientated above the long β-sheets of MERS-CoV. The remodelled structure of SARS-CoV-2 (magenta) has longer β-sheets, and its loop region is more closely aligned with the β-sheets of MERS-CoV.

The structure of the RBD of SARS-CoV-2 was remodelled with Rosetta Comparative Modelling (CM) [19] using the structure of the RBD of MERS-CoV (PDB entry 4L72B) [6] as the template to create an alternative conformation for docking purposes. Sequence and structure alignments were carried out using Promals3D [20]. One thousand structural models were generated, and the best model was selected based upon visual inspection of similarities of the RBDs of SARS-CoV-2 and MERS-CoV (Figure 7). The RBD has been previously defined as the residue range 438–506 [3]. Only the interaction between residue F392 of SARS-CoV-2 and residue I285 of DPP4 lies outside of this domain. Within the RBD, the residue ranges for the anti-parallel β-sheet are 438–442 and 478–481.

### 4.4. Molecular Docking Simulations

HADDOCK 2.4 was used for molecular docking because it can simulate conformational flexibility in the main chains and side chains of the interacting proteins [21]. This was crucial as an important loop in the crystal structure of the RBD of SARS-CoV-2 was predicted to have steric clashes with the hydrophobic binding region of DPP4. Incorporation of molecular flexibility aimed to retain the same binding position observed for the RBD of MERS-CoV with respect to DPP4, but with a different loop conformation at the RBD of SARS-CoV-2. ZDOCK, which utilises a rigid molecular docking approach [20], was also used for the purpose of replicating the predictions of Li et al. [5].

The crystal structures of the RBD of SARS-CoV-2 (PDB entry 6M0JE) [3] and DPP4 (PDB entry 4L72A) [6] were submitted to ZDOCK using the DPP4 residues K267, Q286, T288, A289, R317, R336, Q344, I346, H345 and K392, specified by Li et al. as binding site residues [5]. These authors did not use residues for the RBD of SARS-CoV-2 in their restraints [5]. A total of 54,000 docking poses were generated by ZDOCK, which were then clustered and refined using RDOCK [22].

The crystal structures of the RBD of MERS-CoV (PDB entry 4L72B) [6] and DPP4 (PDB entry 4L72A) [6] were also docked using HADDOCK 2.4 [21]. This was performed as a control prediction to confirm that this molecular docking method could correctly reproduce the crystal structure of the complex of these two proteins. ZDOCK was also used to dock these two proteins [22]. The residues in both proteins used to constrain the docking search are listed in Supplementary Table S1.

In addition, the crystal structures of the RBD of SARS-CoV-2 (PDB entry 6M0JE) [3] and ACE2 (PDB entry 6M0JA) [3] were docked using HADDOCK 2.4 as a further control. The residues used to constrain the docking search are listed in Supplementary Table S2 [3].

Supplementary Table S3 lists the residues in the SARS-CoV-2 remodelled structure that were predicted to interact with DPP4. The same residues in DPP4 were used as unpaired restraints for flexible docking with the crystal structure of SARS-CoV-2.

Three variations with different docking restraints were used for the flexible docking simulations with the crystal structure of the RBD of SARS-CoV-2 using HADDOCK (summarised in Supplementary Table S4). The first variation (CS1) attempted to replicate the approach followed by Li et al. by only specifying the DPP4 residues K267, R336, R317, Q344, Q286 and T288 that these authors defined as key contact residues in their study [5]. The residue Q498 in SARS-CoV-2 was the only restraint specified for this protein and was arbitrarily chosen because Li et al. indicated that it was a residue that interacted with K267 in DPP4 [5], which is known to be a key residue to also bind to MERS-CoV [6,7]. This was necessary because HADDOCK requires residues to be specified for both proteins. This potential residue interaction has been consistently observed across these docking simulations.

The second variation (CS2) again only specified DPP4 residues and residue Q498 in the crystal structure of SARS-CoV-2, but instead used a different list of DPP4 residues: K267, R336, R317, Q344, L294 and I295 [3,5,6,7]. These residues were selected because they were consistently described as key binding residues in the MERS-CoV-DPP4 crystal structure in the literature [6,7]. These are the DPP4 residues that were used henceforth for all other docking simulations.

The third variation (CS3) used the same DPP4 residues specified in the second variation; however, instead of specifying only Q498 for SARS-CoV-2, a larger set of SARS-CoV-2 residues were specified as restraints (see Supplementary Table S4). It is important to note that these residues in the SARS-CoV-2 crystal structure correspond to the residues chosen for the remodelled structure (see Supplementary Table S3). Since the crystal structure of SARS-CoV-2 is in a complex with ACE2, its conformation is unlikely to be suitable for interaction with a different receptor, such as DPP4. In order to remove this bias but still perform molecular docking with the crystal structure, the predicted interactions between DPP4 and SARS-CoV-2 were based upon the SARS-CoV-2 remodelled structure. Consequently, the predicted SARS-CoV-2 residues for the remodelled structure were applied with the SARS-CoV-2 crystal structure as the conformation of the RBD may need to change to bind to DPP4 with a similar binding mode to that with MERS-CoV.

The remodelled structure of the RBD of SARS-CoV-2 was also docked to DPP4 using flexible docking with HADDOCK 2.4. The restraints used were the same used for CS3 above, as listed in Supplementary Table S4.

## 5. Conclusions

Molecular docking simulations were used to try to predict the potential binding interactions between DPP4 and the RBD of SARS-CoV-2. The most successful approach required remodelling of the conformation of the RBD of SARS-CoV-2 to better resemble the conformation of the RBD of MERS-CoV observed in its experimentally determined complex with DPP4. A range of polar and hydrophobic interactions could be predicted; however, the associated predicted binding pose of the RBD of SARS-CoV-2 exhibited important shifts in position compared to the interaction observed in the MERS-CoV-DPP4 complex. The predicted binding energy of interaction was substantially worse with SARS-CoV-2, strongly suggesting that DPP4 is not a likely receptor for this virus. This is consistent with recent biophysical experiments.

## Figures and Tables

**Table 1:** Binding energies of the best docking poses in the MERS-CoV-DPP4, SARS-CoV-2-DPP4 and SARS-CoV-2-ACE2 complexes predicted by HADDOCK. All energies are reported in kJ/mol.

Energy Component	MERS-CoV-DPP4	SARS-CoV-2-DPP4	SARS-CoV-2-ACE2
Van der Waals	−81.8	−94.8	−78.4
Electrostatic	−416.5	−251.8	−230.8
De-solvation	−11.8	−7.9	−24.2
Buried surface area	2602.7	2544.0	2063.5
Binding energy	−384.7	−240.9	−264.5

## Data Availability

Not applicable.

## Supplementary Materials

The following are available online at https://www.mdpi.com/article/10.3390/ijms22137001/s1.
