# Supplementary material for: Does the SARS-CoV-2 Spike Protein Receptor Binding Domain Interact Effectively with the DPP4 (CD26) Receptor? A Molecular Docking Study
Source: Int J Mol Sci. 2021 Jun 29;22(13):7001. doi: 10.3390/ijms22137001 (PMC8269389; doi:10.3390/ijms22137001)
Supplement: Supplementary file 1 [file ijms-22-07001-s001.zip › ijms-1282202-supplementary.pdf]

Does the SARS-CoV-2 spike protein receptor binding domain interact effectively with the DPP4 (CD26) receptor? A molecular docking study.

Kirsten Cameron,[1] Lina Rozano,[2] Marco Falasca[2], and Ricardo L. Mancera[2]*

[1] *School of Molecular and Life Sciences, Curtin University, GPO Box U1987, Perth WA 6845, Australia.*

[2] *Curtin Medical School, Curtin Health Innovation Research Institute and Curtin Institute for Computation, Curtin University, GPO Box U1987, Perth WA 6845, Australia*

* Author for correspondence: R.Mancera@curtin.edu.au.

Supplementary Material

```
                                   10         20         30         40         50         60
                           ....|....| ....|....| ....|....| ....|....| ....|....| ....|....|
6M0JE | SARS-CoV-2 RBD  1   RVQPTESIVR FPNITNLCPF GEVFNATRFA SVYAWNRKRI SNCVADYSVL YNSASFSTFK
4L72B | MERS-CoV RBD    1   ---------- ------ECDF SPLLSGT-PP QVYNFKRLVF TNCNYNLTKL LSLFSVNDFT

                                   70         80         90        100        110        120
                           ....|....| ....|....| ....|....| ....|....| ....|....| ....|....|
6M0JE | SARS-CoV-2 RBD  61  CYGVSPTKLN DLCFTNVYAD SFVIRGDEVR QIAPGQTGKI ADYNYKLPDD FTGCVIAWNS
4L72B | MERS-CoV RBD    44  CSQISPAAIA SNCYSSLILD YFSYPLSMKS DLSVSSAGPI SQFNYKQSFS NPTCLILATV

                                  130        140        150        160        170        180
                           ....|....| ....|....| ....|....| ....|....| ....|....| ....|....|
6M0JE | SARS-CoV-2 RBD  121 NNLDSKVGGN YNYLYRLFRK SNLKPFERDI STEIYQAGST PCNGVEGFNC YFPLQSYGFQ
4L72B | MERS-CoV RBD    104 PHNLTTITKP LKYSYINKCS RLLSDDRTEV PQLVNANQYS PCVSIVPSTV WE--------

                                  190        200        210        220        230
                           ....|....| ....|....| ....|....| ....|....| ....|....| .
6M0JE | SARS-CoV-2 RBD  181 PTNGVGYQPY RVVVLSFELL HAPATVCGPK KSTNLVKNKC VNFHHHHHH- -
4L72B | MERS-CoV RBD    155 --DGDYYRKQ LSPLEGGGWL VASGSTVAMT EQLQMGFGIT VQYGTDTNSV C
```

Figure S1: Sequence alignment of the RBDs in MERS-CoV and SARS-CoV-2. For SARS-CoV-2, the start of this sequence corresponds to residue 319 in the crystal structure [2]. For MERS-CoV, the start of this sequence corresponds to residue 382 in the crystal structure [6].

```
MERS_4L72B     382 ECDFSPLLSGTPPQVYNFKRLVFTN-CNYNL--------TKLLSLFSVNDF
SARS2_6M0JE    343 NATR-------FASVYAWNRKRISN-CVADYSVLYNSAS--------FSTF
SARS2_Remodel  331 ---------NITNLCPFGEVFNATRF-ASVY--------AWNRKRISNCVA
                            ................ .....        ............

MERS_4L72B     424 TCSQISPAAIASNCYSSLILDYFSYPLSMKSDLSVSS-AGPISQFNYKQS-
SARS2_6M0JE    378 KCYGVSPTKLNDLCFTNVYADSFVIRGDEVRQIAPGQ-TGKIADYNYKLPD
SARS2_Remodel  364 DYSVLYNSASFSTFKCYGVSPTKLNDLCFTNVYADSFV-IRGDEVRQIAP-
                   ................................. ...........

MERS_4L72B     473 --FSNPTCLILAT--------------------------------------
SARS2_6M0JE    428 DF---TGCVIAWNSNNLDSKVGGNYNYLYRLFRKSNLKPFERDISTEIYQA
SARS2_Remodel  413 --GQTGKIADYNY--------------------------------------
                     ...........

MERS_4L72B         --------------------------------------------------
SARS2_6M0JE    476 GSTPCNGVEGFNCYFPLQSYGFQPTNGVGYQPYRVVVLSFELLHAPATVCG
SARS2_Remodel      --------------------------------------------------

MERS_4L72B     484 VPHNLTTITKPLKYSYINKCSRLLSDDRTEVPQLVNANQYSPCVSIVPSTV
SARS2_6M0JE        --------------------------------------------------
SARS2_Remodel  424 KLPDDFTGCVIAWNSNNLDSKVGGNYNYLYRLFRKSNLKPFERDISTEIYQ
                   ....   ..   ........

MERS_4L72B     535 WEDGDYYRKQLSPLEGGGWLVASGSTVAMTEQLQMGFGITVQYGTDTNSVC
SARS2_6M0JE        --------------------------------------------------
SARS2_Remodel  475 AGSTPCNGVEGFNCYFPLQSYGFQPTNGVGYQPYRVVVLSFELLHAPATVC

MERS_4L72B         ---------------------
SARS2_6M0JE        ---------------------
SARS2_Remodel  526 GPKKSTNLVKNKCVNFHHHHHH
```

Figure S2: Structural alignment of the RBDs of MERS-CoV, SARS-CoV-2 and the remodelled SARS-CoV-2 structure. Structural alignment is indicated by dots underlying the three sequences.

Table S1: Amino acid residues used for docking restraints for MERS-CoV and DPP4. The MERS-CoV residues that are known to interact with a specific DPP4 residue are listed in the rows corresponding to that DPP4 residue; however, these were not submitted as paired interaction docking restraints.

DPP4	MERS-CoV
L294	V555
I295	W553, L506
Q344	E513
R317	D510
R336	Y499
K267	D539

Table S2: Amino acid residues used for docking restraints in HADDOCK for SARS-CoV-2 and ACE2. These residues were not submitted as paired docking restraints.

ACE2	SARS-CoV-2
D30	K417
E35	Q493
Y41	N501
Q24	N487
Q42	G446
D38	Y499
E37	Y505
Y41	T500
G354	N501
K353	G502
K31	G496
M82	F486
Y83	F456
H34	L455
Q42	Y449
T27	Y453
F28	A475
N330	
D355	
R357	
R393	

Table S3: Amino acid residues specified as the parameters for flexible docking using HADDOCK for DPP4 and the remodelled structure of the RBD of SARS-CoV-2. The amino acid restraints for each SARS-CoV-2 structure and DPP4 were submitted as unpaired interaction docking restraints. Each DPP4 residue had one or more SARS-CoV-2 residues predicted to have a potential interaction.

DPP4	SARS-CoV-2 remodelled structure
K267	N481
	T478
R336	N439
R317	—
Q344	K444
L294	L461
I295	Y473

Table S4: Amino acid residues specified as the docking restraints in the crystal structure of the RBD of SARS-CoV-2. CS1, CS2 and CS3 refer to the first, second and third variations in docking restraints, respectively, as described in the main text.

CS1		CS2		CS3	
**DPP4**	**SARS-CoV-2**	**DPP4**	**SARS-CoV-2**	**DPP4**	**SARS-CoV-2**
K267	Q498	K267	Q498	K267	N439
					T478
R336		R336		R336	N481
R317		R317		R317	-
Q344		Q344		Q344	K444
Q268		L294		L294	L461
T288		I295		I295	Y473

Table S5: Comparison of the binding interactions and interacting residues between the RBD of MERS-CoV and DPP4 reported experimentally (crystal structure) and predicted by HADDOCK.

	**CRYSTAL STRUCTURE**		**HADDOCK**	
**Type of interaction**	**DPP4**	**MERS-CoV**	**DPP4**	**MERS-CoV**
Salt bridge and H-bond	K267	D539	K267	D539<br>G538
H-bond	R336	Y499	R336	Y499
H-bond	Q344	E513	Q344	E513
Salt bridge	R317	D510	R317	D510
Hydrophobic	L294	V555	L294	V555
Hydrophobic	I295	L506<br>W553	I295	L506
Hydrophobic			V341	P515
H-bond			Q286	N501
H-bond			S334	S454
H-bond			D331	A461

Table S6: Energies of binding of the best docking pose in the three docking variation restraints of the interaction of the RBD of SARS-CoV-2 with DPP4 predicted by HADDOCK. All energies are reported in kJ/mol.

**Energy component**	**CS1**	**CS2**	**CS3**
Van der Waals	-75.4	-53.3	-44.4
Electrostatic	176.5	-153.5	-156.5
Desolvation	-12.2	-6.7	-2.3
Buried surface area	1685.5	1886.1	1754.0
Binding energy	-70.0	-121.5	-114.6
